# Lower cervical C6/C7 andersson lesion with upper cervical C1/C2 fracture in ankylosing spondylitis: a case report and literature review

**DOI:** 10.3389/fsurg.2025.1568553

**Published:** 2025-05-27

**Authors:** Han Qiao, Xiaofei Cheng, Haijun Tian, Changqing Zhao, Xiaojiang Sun, Jie Zhao

**Affiliations:** ^1^ Department of Orthopaedic Surgery, Shanghai Ninth People’s Hospital, Shanghai Jiao Tong University School of Medicine, Shanghai, China; ^2^ Shanghai Key Laboratory of Orthopaedic Implants, Shanghai Ninth People’s Hospital, Shanghai Jiao Tong University School of Medicine, Shanghai, China; ^3^ Department of Orthopaedic Surgery, Shanghai Sixth People’s Hospital, Shanghai Jiao Tong University School of Medicine, Shanghai, China; ^4^ Department of Orthopaedic Surgery, Fengcheng Hospital of Fengxian District, Shanghai, China

**Keywords:** andersson lesion, ankylosing spondylitis, atlantoaxial fracture, spinal stability, cervical surgery

## Abstract

Cervical andersson lesions (ALs) are rare in patients with ankylosing spondylitis (AS), and even more rare in patients with simultaneous superior cervical atlantoaxial fracture and dislocation. Here, we present a case of C1 Jefferson fracture (C1 bilateral posterior arch fracture), C2 odontoid, lateral mass, vertebral fracture (nonclassic C2 hangman fracture), traumatic posterior atlantoaxial dislocation (AAD) and C6/C7 AL in a long-standing AS cervical spine. The patient with traumatic AS-related cervical fractures underwent a two-stage surgery. The stage I surgery involved a posterior atlantoaxial reduction and fixation surgery combined with C5/C6/T1/T2 posterior pedicle screw fixation plus C6/C7 decompression. One week later, C6/C7 anterior cervical corpectomy decompression and fusion (ACCF) with long anterior plate stabilization combined with iliac crest bone graft transplantation was performed for stage II surgery. The patient recovery observed during follow-up was satisfactory. Nine-month postoperative radiological images revealed fracture union of the upper and lower cervical spine with optimal reduction of the atlantoaxial segment. In conclusion, lower cervical ALs with simultaneous upper cervical C1/C2 fractures in the AS are very rare. Posterior C1-C2 fixation combined with C6-C7 AL corpectomy/fusion and posterior pedicle screw fixation may offer a desirable alternative approach for this complex case of cervical trauma. During treatment, complete decompression, effective reduction, and potent stabilization can comprehensively improve the clinical prognosis.

## Introduction

Ankylosing spondylitis (AS) is characterized by inflammatory ossification of the fusion between the sacroiliac joint and ankylosed spinal ligaments and presents as a stiff spine and osteoporosis (1). Therefore, patients with AS inevitably incur spinal fractures, which can be caused by even trivial traumatic impacts. Andersson lesions (ALs), known as erosive vertebral or discovertebral lesions in AS, are characterized by consistent spinal fracture nonunion, spondylodiscitis and pseudarthrosis (2). ALs are commonly found in the thoracic and lumbar regions of the spine rather than in the lower cervical spine (3, 4). This was partially due to the reduced mechanical stress in the cervical spine compared with that in the thoracolumbar skeleton. In addition, acute injury to the C1/C2 complex poses a potentially life-threatening challenge to the spine cord, resulting in severe neurological sequelae derived from unstable upper atlantoaxial joints. Very few studies have reported upper C1/C2 trauma with AS (5–7), and there are even few cases in which lower AL simultaneously presents with upper atlantoaxial fracture in a single patient. Herein, we describe the case in which a patient with AS was sent to our hospital after falling from a motorcycle during a collision. CT revealed a C1 Jefferson fracture (C1 bilateral posterior arch fracture), C2 odontoid fracture, lateral mass, vertebral fracture (nonclassic C2 hangman fracture), traumatic posterior atlantoaxial dislocation (AAD) and C6/C7 AL in the long-standing AS spine. Upon further history questioning, the patient recalled persistent neck pain after a previous fall two years ago but did not previously seek medical assistance. To the best of our knowledge, this is the first report that covers both the lower C6/C7 AL and upper C1/C2 AL fractures in the ankylosed spine. We aimed to identify the significant correlation between spinal stability and both C1/C2 and C6/C7 injuries by elucidating their clinical and treatment characteristics, aiming to provide a deeper understanding of cervical spine stability, especially in patients with AS.

### Patient characteristics

#### Descriptions

A 49-year-old man experienced occipitocervical junction pain with restricted cervical range of motion while falling during a motorcycle collision. Significant numbness was found in both upper limbs, with reduced bilateral biceps and intrinsic hand muscle strength, especially in the left upper arm. The Lhermitte sign was positive when he bent his neck or rolled over on both sides, radiating from the neck to the planta pedis. The diagnosis of AS was established five years prior, but the patient failed to take proper medication regularly. He had experienced an accident two years prior, which was followed by consistent neck discomfort. However, he did not seek medical assistance during that period and played basketball several times.

**Figure 1:**
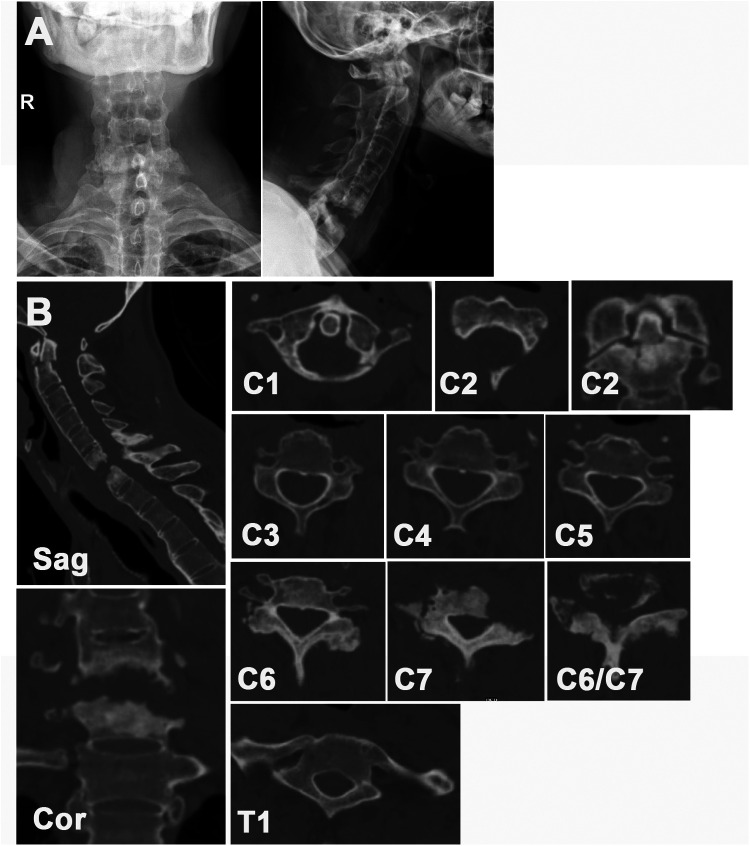
**(A)** preoperative cervical x-ray image. **(B)** Preoperative cervical CT image.

**Figure 2:**
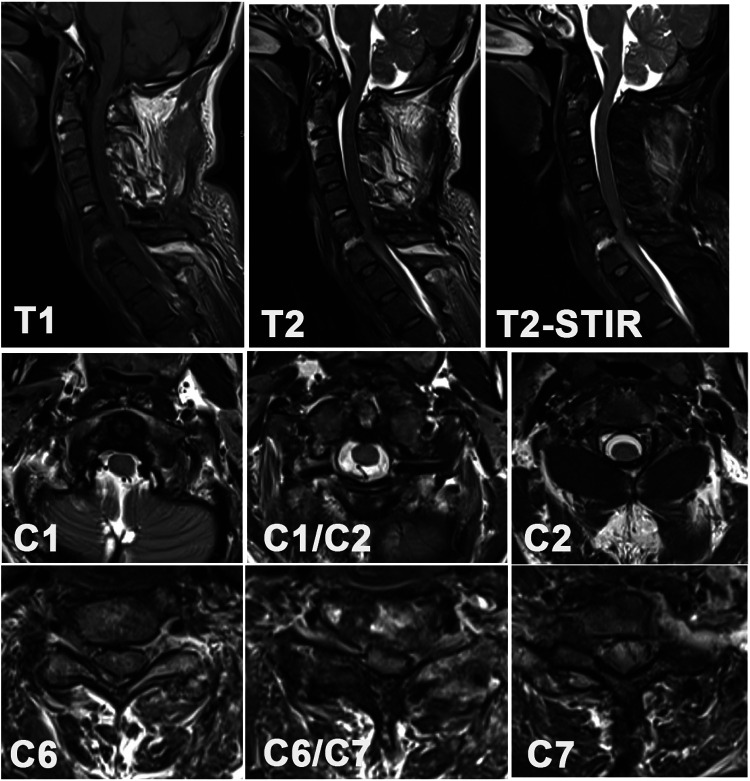
Preoperative cervical MRI.

The characteristics of the patient are listed in Tables 1–3. The x-ray image revealed a bamboo-like ankylosed cervical spine (Figure 1A). A CT scan revealed the detailed characteristics of both the superior and inferior cervical fractures (Figure 1B). The C1 fracture was identified as a Jefferson fracture with bilateral posterior arch fracture, which was classified as type II Levine-Edwards classification with traumatic posterior AAD. The C2 vertebra suffers from odontoid fracture (type IIB Andersson-D'Alonzo classification) and lateral mass fracture of the vertebral wall (nonclassic C2 hangman fracture (8). Herein, the C6/C7 segment exhibited osteolysis combined with sclerosis in the upper and lower endplates, with the disappearance of the normal C6/C7 intervertebral structure. This process was followed by further destruction of the C6/C7 bony vertebrae. MRI (Figure 2) revealed vertebral edema in the C2 odontoid and vertebral walls. Additionally, the increase in the T2 signal was concentrated at the C6/C7 intervertebral level. The spinous process and supraspinous ligaments were injured, which was exemplified by the enhanced T2 intensity from C2 to the C7 posterior ligament complex (PLC). More importantly, the stenotic spinal cord was evidently compressed in the C6/C7 segment with diffuse intradural hyperintensity, which explained the reduced muscular strength and sensation in the extremities.

**Table 1:** Clinical characteristics.

Category	Details
General info	
Age	49
Gender	Male
Fracture classification	
C1 fracture	Type II Levine-Edwards classification
C2 odontoid fracture	Type IIB Andersson-D'Alonzo classification
Reducible	Yes
AAD	Posterior
Neurological status	
Lhermitter sign	Positive
Hoffmann sign	Negative
Babinski sign	Negative

**Table 2:** Cervical neurological examination for sensation.

Time point	Pre-1st OP				Pre-2nd OP				Post-OP^a^				9 m post-OP			
mJOA	7				8				6				16			
VAS	8				6				6				1			
Location	Left		Right		Left		Right		Left		Right		Left		Right	
Nerve	P	T	P	T	P	T	P	T	P	T	P	T	P	T	P	T
C2	2	2	2	2	2	2	2	2	2	2	2	2	2	2	2	2
C3	2	2	2	2	2	2	2	2	2	2	2	2	2	2	2	2
C4	2	2	2	2	2	2	2	2	2	2	2	2	2	2	2	2
C5	1	1	1	1	1	1	1	1	1	1	1	1	2	2	2	2
C6	1	1	1	1	1	1	1	1	1	1	1	1	2	2	2	2
C7	1	1	1	1	1	1	1	1	1	1	1	1	2	2	2	2
C8	1	1	1	1	1	1	1	1	1	1	1	1	2	2	2	2
T1–T12	1	1	1	1	1	1	1	1	1	1	1	1	2	2	2	2
L1–L5	1	1	1	1	1	1	1	1	1	1	1	1	2	2	2	2
S1–S5	1	1	1	1	1	1	1	1	1	1	1	1	2	2	2	2

P, pain; T, tactual.

2 stands for normal, 1 stands for decrease, 0 stands for disappear.

^a^ Post-OP: instantly after second surgery.

**Table 3:** Neurological examination for MRC.^a^

Time point	Pre-1st OP		Pre-2nd OP		Post-OP^b^		Post-OP 9 m	
Nerve	Left	Right	Left	Right	Left	Right	Left	Right
C4	4	4	5	5	5	5	5	5
C5	3	3	4	4	4	4	5	5
C6	3	3	4	4	3	3	5	5
C7	3	3	4	4	3	3	5	5
C8	2	3	3	3	3	3	5	5
L2	3	3	4	4	3	2	5	5
L3	3	3	4	4	3	2	5	5
L4	3	3	4	4	3	2	5	5
L5	3	3	4	4	3	2	5	5
S1	3	3	4	4	3	2	5	5

^a^ MRC scale, medical research council scale for muscular strength.

^b^ Post-OP: instantly after second surgery.

#### Surgery

Because this patient suffered from two separate traumas that contributed to the C1/C2 fracture and C6/C7 AL, both superior and inferior cervical lesions required sufficient decompression to rescue neurologic deficits as well as potent fixation to restore spinal stability via two sequential surgical operations. The initial surgery was posterior atlantoaxial reduction and fixation surgery combined with C5/C6/T1/T2 posterior pedicle screw fixation plus C6/C7 decompression. One week later, C6/C7 anterior cervical corpectomy decompression and fusion (ACCF) with long anterior plate stabilization combined with iliac crest bone graft transplantation was performed as a second surgery.

After the first posterior surgery, the patient experienced rapid neurologic recovery of limb numbness and weakness compared with his preoperative symptoms. However, after the second anterior surgery, the patient experienced a transient decrease in limb muscular strength, especially in the right lower limb (2/5). We considered that during the second C6/C7 ACCF surgery, because there was a large amount of fibrous scar tissue in the C6/C7 intervertebral space, which was accompanied by an unclear intervertebral structure and the formation of numerous osteophytes in the front edge of the adjacent upper and lower vertebral endplates, no obvious bony structures were observed in the posterior wall of the C6 and C7 vertebral bodies. The scar fibrous tissue was closely adherent to the dural sac and was not clearly and thoroughly separated. Hence, during the decompression process, inevitable disturbance may occur to the spinal cord, which can lead to the transient aggravation of neurologic symptoms. Subsequently, the patient received low-dose methylprednisolone and mannitol dehydration treatment. The patient's neurologic symptoms significantly improved within three days. His muscular strength of the left lower limb increased to 4/5. During the follow-up, he received regular NSAIDs treatment.

**Figure 3:**
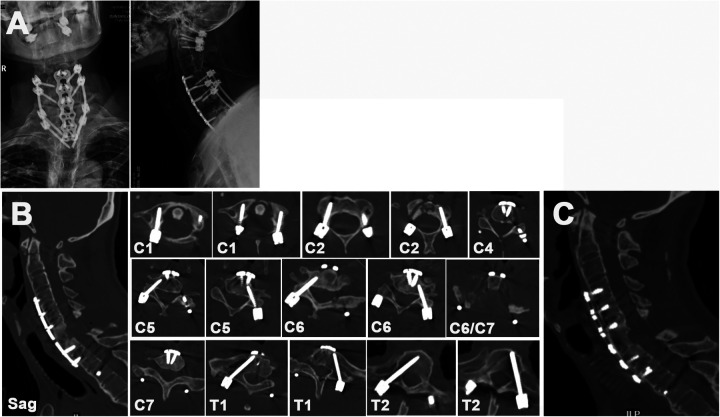
**(A)** postoperative cervical x-ray image. **(B)** Postoperative cervical CT image 2 days after surgery. **(C)** Postoperative cervical sagittal CT image 9 months after surgery showing healing of the odontoid fracture and bone fusion after C6 iliac bone transplantation.

The final postoperative x-ray is shown in Figure 3A. Postoperative CT images (Figure 3B) demonstrated the precise implantation of each pedicle screw and the stable support of the anterior iliac crest bone graft. Figure 3C shows the bone healing observed after 9 months of follow-up. The AAD was corrected, and the stenotic C6/C7 lesion was decompressed sufficiently. After nine months of follow-up, the patient remained neurologically intact with no evident neck or limb discomfort. No other complication occurred during the rest follow-up period. He was able to walk freely without any brace help. The postoperative VAS score was 1, and the mJOA score was 16 compared with 8 and 7 preoperatively.

## Discussion

The unique feature of our patient was that he incurred two significant traumas. After the first trauma, cervical spinal stability was maintained partially by relying on fibrous union until the second severe trauma occurred two years later. These two separate falls cause lower and upper cervical fractures, respectively. Approximately 14% of AS patients clinically suffer from AS fracture (9), while only 1.5% progress to persistent AL bone nonunion (10). Altered biomechanics increase the risk of spinal fracture, thereby contributing to the progression of AL by persistently increasing vertebral/discovertebral stress and stimulating discitis/spondylodiscitis (11). Herein, the etiologies of AL varied. It can be classified as traumatic or inflammatory, which manifests as early spondylodiscitis or late pseudarthrosis, respectively (3). More importantly, the rigidity and fragility of the fused AS spine can act as a brittle plaster rather than an elastic spring, which is especially vulnerable to the uneven distribution of mechanical impact (12, 13). Therefore, even trivial mechanical stress can lead to severe loss of spinal stability, with 58% of patients being diagnosed with devastating neurologic sequalae (14). Spinal cord injury can be caused by instability of the cervical spine or direct stenotic compression of the bone fracture. Surgical approaches were thereby employed to resolve the spinal cord stimulation, reestablish the spinal curvature and restore normal neurologic functionality (15). Herein, preoperative MR revealed significant spinal cord compression at the stenotic C1/C2 and C6/C7 levels, indicating that the proper reduction and fixation of the responsible segments was indispensable for alleviating clinical symptoms. With regards to the AL, we chose C6/C7 ACCF with long anterior plate stabilization and iliac crest bone graft transplantation to debride the proliferative inflammatory tissue and reconstruct the immobilizer vertebral column. Additionally, posterior C5-T2 pedicle screw fixation assisted in maintaining cervical spine stability.

The common site of AL in AS patients is the thoracic and lumbar spine, especially in the thoracolumbar transition area (T10-L2) (16, 17); few studies have reported cervical AL, which is relatively rare (18), not to mention the concomitant occurrence of both cervical AL and C1 Jefferson fractures and C2 odontoid fractures. Peng (18) reported a 78-year-old AS patient suffered from C5/6 and C6/7 AL who received one stage anterior-posterior approach. The follow-up results showed the desirable relief of limb pain and movement. Since both C1–C2 dislocation and C6/C7 AL contributed to severe cervical instability, the use of a nonoperative treatment algorithm was not sufficient to correct the dynamic cervical imbalance. To date, numerous treatment modalities are used for restoring the cervical spine. Previously, transoral decompression, followed by posterior fixation or anterior fixation, was used to manage cervical sagittal alignment (19). However, due to the presence of multiple surgical incisions and the associated risk of infection, this combined surgical approach is not frequently employed in clinical practice. Eun (20) et al. presented a case of type II odontoid fracture with AAD in an AS patient who was treated with cervical reduction and occipito-cervical fusion (OCF) from C0-C4. Prognosis evaluation showed the satisfied recovery at 3 years postoperatively. Miao et al. also reported a series of cases of C2 odontoid fracture of the AS spine with posterior OCF and autologous bone transplantation, showing that the fracture union and dislocation were reduced (5). Mika et al. (21) revisited the MRI results of 20 AS cervical fracture patients and showed that only two patients suffered from both odontoid fracture and C1 Jefferson fracture. However, the researchers did not mention their corresponding treatment approaches. In addition, anterior odontoid screw fixation was employed to restore the cervical curvature in C2 odontoid fracture patients with AS, demonstrating the relief of neck symptoms with the retention of neck rotation functionality (22). Therefore, the surgical approach for treating cervical fractures in AS patients is controversial. Compared with lateral mass screws, posterior C1/C2 pedicle screw fixation for atlantoaxial immobilization can significantly decrease the postoperative NDI score with more efficient anti-pullout efficacy (23). More importantly, posterior C1/C2 pedicle screw fixation can be removed after fracture union, leading to the effective retention of neck rotary function. Compared with C1/C2 fixation without fusion, conventional C1/C2 fixation and iliac bone transplantation/fusion significantly reduced atlantoaxial rotation, leading to incongruous neck stiffness and discomfort (24). Additionally, anterior C1/C2 fixation was not applicable in our case of atlantoaxial fracture. The appropriate placement of anterior odontoid screws was challenging due to the traumatic kyphosis of the cervical spine as well as the osseous anterior longitudinal ligament (ALL). In contrast, with proper operative traction and reduction, posterior atlantoaxial fixation and fusion can satisfactorily meet the requirements for C1/C2 fracture healing (25). However, whether OCF with long-segment fixation or C1/C2 fusion with short-segment fixation results in atlantoaxial fracture of the AS spine remains debatable. Miao et al. (5) showed that OCF from C0-C4 posterior fixation can result in optimal atlantoaxial fracture union. This approach was not applicable in our patient due to the lower C6/C7 AL. The potential long-segment fixation from C0-C4 may have led to the new initiation of AL in the transition area between C0-C4 and C5-T2 after even trivial trauma. More importantly, the application of osteogenic agents and anti-osteoporosis remedies may serve as potent accelerators for bone fracture healing (26). Hence, individualized surgical fracture treatment is recommended based on diverse traumatic patterns, bone density and surgeon judgment.

Herein, our manuscript is the first to describe the combined occurrence of both upper C1/C2 fractures and AADs with C6/C7 ALs in AS patients. In addition, subsequent cervical reduction and posterior/anterior fixation and fusion resulted in desirable relief of cervical symptoms, as indicated by improvements in the VAS and mJOA scores. Our case provides an essential reference for multiple cervical injuries in AS patients. Extensive stenotic decompression and robust cervical stability are crucial for the treatment of multiple cervical traumas, especially in AS. However, when performing the fixation approach and fusion segment selection, various aspects should be considered individually and comprehensively.

## Conclusion

Our review revealed a combination of superior atlantoaxial fracture (C1 Jefferson fracture and C2 type II odontoid fracture) with an inferior C6/C7 Andersson lesion in the rigid spine of the AS. Posterior C1/C2 fixation combined with C6/C7 AL corpectomy/fusion and posterior pedicle screw fixation could serve as a desirable alternative approach for this complex case of cervical trauma. During treatment, complete decompression, effective reduction, and potent stabilization can comprehensively improve the clinical prognosis.

## Data Availability

The raw data supporting the conclusions of this article will be made available by the authors, without undue reservation.
